# Detection of *GNAS* mutations in intramuscular / cellular myxomas as diagnostic tool in the classification of myxoid soft tissue tumors

**DOI:** 10.1186/s13000-018-0734-8

**Published:** 2018-08-15

**Authors:** Sandra Sunitsch, Magdalena Maria Gilg, Karl Kashofer, Franz Gollowitsch, Andreas Leithner, Bernadette Liegl-Atzwanger

**Affiliations:** 1 Institute of Pathology, Klinikum Klagenfurt am Wörthersee, Feschnigstraße 11, 9020 Klagenfurt am Wörthersee, Austria; 2 Institute of Pathology, Medical University of Graz, Neue Stiftingtalstraße 6, 8010 Graz, Austria; 3 Department of Orthopedics and Trauma Surgery, Medical University of Graz, Auenbruggerplatz 5, 8036 Graz, Austria

**Keywords:** Soft tissue pathology, Intramuscular / cellular myxoma, Myxofibrosarcoma, *GNAS* mutation

## Abstract

**Background:**

Intramuscular / cellular myxomas and low-grade myxofibrosarcomas are two different tumor entities with a significant histological overlap, especially if dealing with small biopsies. Despite the morphological similarities, they differ considerably in their biological behaviour. Intramuscular / cellular myxoma rarely shows signs of recurrence and never metastasizes, in contrast to myxofibrosarcoma that tends to recur more aggressively and to metastasize haematologically. Therefore, it is of great importance to distinguish these lesions - evaluation of *GNAS* mutation status could be of tremendous help.

**Methods:**

We reviewed 13 cases with intramuscular / cellular myxomas. The 13 cases included 5 men and 8 women, aged from 33 to 71 years (mean age 55.5 years). Immunohistochemistry was performed as well as next generation sequencing. Ten cases were located in the lower extremities and three cases were located in the upper extremities. Two lesions were initially misdiagnosed as a low-grade myxofibrosarcoma.

**Results:**

Performing next generation sequencing 12 out of 13 specimens showed a *GNAS* mutation.

**Conclusions:**

Our findings demonstrate that *GNAS* mutations are more common in intramuscular / cellular myxomas, than had been reported in literature in the past. Next generation sequencing for determining *GNAS* mutation status on small biopsies or diagnostically challenging cases facilitates the diagnosis of intramuscular / cellular myxoma and separates this tumor entity from its mimics.

## Background

Myxoid soft tissue tumors are challenging to diagnose, especially on core needle biopsies [1]. Based on tumor heterogeneity, commonly seen in soft tissue tumors, there is a tremendous morphologic overlap between benign and malignant myxoid soft tissue tumors with spindle cell morphology. Age of patients, tumor location, imaging as well as hematoxylin and eosin (HE) morphology in combination with immunohistochemistry can help to classify these tumors properly to allow an adequate treatment [2]. Pathologists are more and more confronted with biopsy specimens and should be aware of the limitations and the diagnostic risks. To be as precise as possible even in small tissue specimens the use of next generation sequencing in combination with HE morphology and immunohistochemistry can clearly facilitate the exact tumor characterization.

Especially differential diagnosis of intramuscular / cellular myxomas and low-grade myxofibrosarcomas is challenging based on similar morphologic features and similarities in clinical presentation [2–7]. Intramuscular / cellular myxoma usually affects middle-aged adults (median age 50–60) with a female predominance and occurs in the lower extremities, especially in the thigh [8]. Most intramuscular / cellular myxomas occur intramuscular and just a small subset of them occur in a subcutaneous location. Histologically, intramuscular myxomas are hypocellular lesions with small bland cells, with oval nuclei, inconspicuous nucleoli and an abundant myxoid stroma. Embedded in the myxoid matrix scattered blood vessels are found [8, 9].

Cellular myxoma has a variable prominent focal or diffuse increase in cellularity. Additionally, accompanied by an increase in vascularity and a collagenous stroma - any apparent cytonuclear atypia or cell-hyperchromasia is not visible [10].

Myxofibrosarcomas are the most common sarcomas of the elderly. These tumors usually occur in the extremities, especially in the thigh. They occur in superficial and deep location and can show an infiltrative growth pattern. An abundant myxoid matrix, prominent curvilinear vessels, and spindled or stellate cells with eosinophilic cytoplasm and hyperchromatic nuclei histologically characterize low-grade myxofibrosarcomas. In low-grade lesions mitotic figures are hardly found [11, 12].

Although these tumors are difficult to distinguish, especially in small biopsy specimen, exact classification is essential for treatment based on the different biological behavior; Low-grade myxofibrosarcomas commonly recur, sometimes even as high-grade sarcoma. Therefore, a wide excision is required for myxofibrosarcoma whereas a marginal excision for intramuscular / cellular myxomas is sufficient [8, 12].

The aim of the study was to evaluate the use of next generation sequencing as diagnostic tool in the evaluation of myxoid soft tissue tumors.

## Methods

Two cases were part of a previous retrospective study on myxofibrosarcomas. These two cases (#1 and #2, Table 1) were initially diagnosed as low-grade myxofibrosarcomas. However, by performing mutational analysis the detected *GNAS* mutation prompted us to reclassify these 2 tumors as cellular / intramuscular myxomas. These two index cases prompted us to start a study on consecutively submitted biopsy specimens of 11 myxoid tumor samples (#3 to #13, Table 1) where the distinction between low-grade myxofibrosarcoma and cellular / intramuscular myxoma could not be reached on morphology. Mutational analysis was performed in these cases as part of the routine diagnostic process.

**Table 1:** Clinicopathological data of our cases

Case	Initial Diagnosis	Age	Sex	Localization	Site	Diameter	*GNAS*	Sample
1	Myxofibrosarcoma G1	< 40	female	Upper arm	deep	3.2 cm	yes	biopsy
2	Myxofibrosarcoma G1	> 60	female	Musculus gluteus medius	deep	8.5 cm	yes	biopsy
3	Intramuscular / cellular myxoma	< 50	female	Musculus soleus	deep	2.6 cm	yes	biopsy
4	Intramuscular / cellular myxoma	< 60	male	Musculus gastrocnemius	deep	3.1 cm	yes	biopsy
5	Intramuscular / cellular myxoma	< 60	male	Musculus vastus lateralis	deep	3.2 cm	yes	resection
6	Intramuscular / cellular myxoma	> 60	male	Thigh	deep	3 cm	yes	biopsy
7	Intramuscular / cellular myxoma	< 50	male	Musculus semimembranosus	deep	7 cm	yes	resection
8	Intramuscular / cellular myxoma	> 60	female	Musculus rectus femoris	deep	4.3 cm	yes	biopsy
9	Intramuscular / cellular myxoma	< 60	male	Upper arm	deep	5 cm	yes	biopsy
10	Intramuscular / cellular myxoma	> 60	female	Thigh	deep	6 cm	yes	biopsy
11	Intramuscular / cellular myxoma	> 60	female	Thigh	deep	7 cm	yes	biopsy
12	Intramuscular / cellular myxoma	< 60	female	Forearm	deep	3 cm	yes	biopsy
13	Intramuscular / cellular myxoma	> 60	female	Thigh	deep	4 cm	no	biopsy

The biopsy specimens were diagnosed between 2011 and 2017.

Formalin fixed and paraffin embedded tissues of 13 patients were routinely processed. Histological sections were stained with HE according to standard protocol. Immunohistochemistry was performed on an Omnis Autostainer with antibodies against pankeratin, CD34, SMA, desmin, EMA, MUC4 and S100 (all Dako). Myxoid soft tissue tumors with recurrent gene fusions (for example: low-grade myxoid liposarcoma, low-grade fibromyxoid sarcoma, myxoinflammatory fibroblastic sarcoma, myxoid dermatofibrosarcoma protuberans…) were excluded from this study.

Next generation sequencing libraries were prepared using the AmpliSeq library kit 2.0 (Thermo Fisher Scientific) and an Ion Ampliseq primer panel covering mutation hotspots in *BRAF*, *CTNNB1*, *GNAS*, *PDGFRA*, *GNAQ*, *MED12*, *MYOD1*, *PIK3CA* as well as *KIT* (exons 8,9,11,12,13,17,18), *TP53* (exons 4–10) and the full coding sequence of *FH*, *PTCH1*, *NF1*, *NF2*, *RB1*, *PRKAR1A*, *TSC1* and *TSC2*. Sequencing was performed to a length of 200 base pairs on an Ion Proton benchtop sequencer (Thermo Fisher Scientific) to yield an average depth of 5000×. Variant calling required a minimum coverage of 100× for mutation detection. Initial data analysis was done using the Ion Torrent Suite Software 5.4 Plug-ins (Thermo Fisher Scientific, open source, GPL, https://github.com/iontorrent/). Briefly, this included base calling, alignment to the reference genome (HG19) using the TMAP mapper and variant calling by a modified diBayes approach taking into account the flow space information. Called variants were annotated using open source software ANNOVAR [13] and SnpEff [14]. All coding, non-synonymous mutations preliminary reported by the variant caller were further evaluated and visually inspected in IGV (http://www.broadinstitute.org/igv/), and variant calls resulting from technical read errors or sequence effects were excluded from the analysis.

## Results

We analyzed 13 patients (5 male, 8 female) with an age range from 33 to 71 years, mean age 55.5 years. To completely anonymize the data, we have excluded the patients age in Table 1 - however we gave specifications < 40, < 50, < 60, > 60. From 11 out of 13 patients, small biopsy specimens were submitted prior to resection. Ten tumors were located in the lower extremities and just three tumors were located in the upper extremities. All tumors showed at least on imaging a good circumscription and were only located in the deep muscle. The tumor size ranged from 2.6 cm to 8.5 cm (Table 1). In all tumors with follow up (9/13) no recurrence or metastases occurred.

**Fig. 1:**
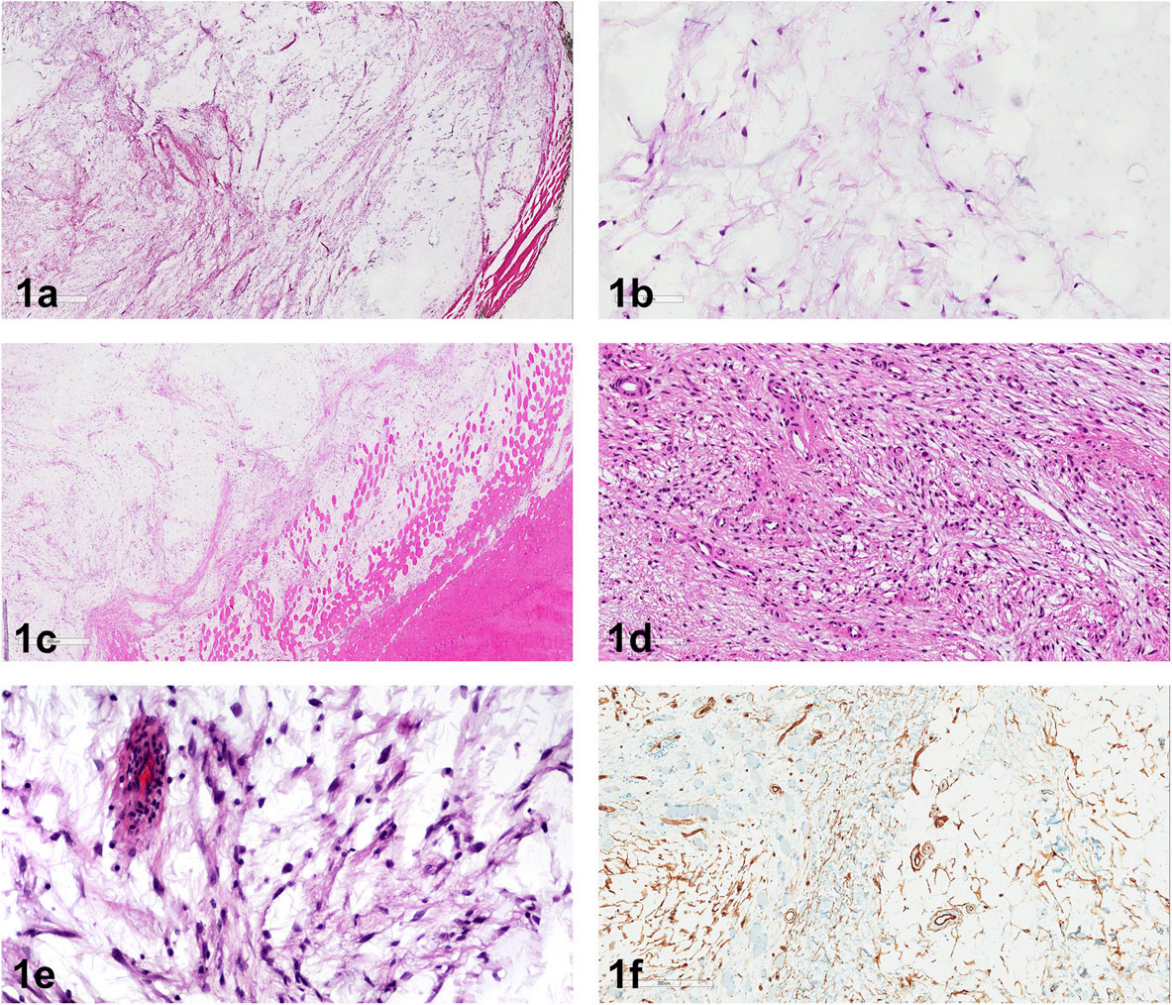
**a** Shows a hypocellular lesion with small bland cells, with oval nuclei and inconspicuous nucleoli and abundant myxoid stroma. **b** Embedded in the myxoid matrix are scattered blood vessels and small bland cells. **c** At the periphery of this lesion muscle fibers are split up by the mucoid matrix. **d** This lesion shows an increase in cellularity, accompanied by an increase in vascularity and collagenous stroma. **e** Area with spindled cells showing cytonuclear atypia. **f** Immunohistochemistry reveals focal CD34 expression

Histologically, all specimens showed uniform, spindled to stellated cells with tapering eosinophilic cytoplasm and in the majority of cases with small unsuspicious nuclei. The cells were embedded in an abundant myxoid matrix containing a vascular network composed of capillary sized blood vessels (Fig. 1a and b). Sometimes curvilinear vessels could be seen. At the periphery, these tumors merged with the surrounding skeletal muscle fibers. Myxoid tumor matrix encased muscle fibers (Fig. 1c). Cellular areas were seen in most of the cases (Fig. 1d), additionally we also could identify areas with spindle cells demonstrating nuclear hyperchromasia (Fig. 1e). Immunohistochemically, diffuse or focal CD34 positivity (cytoplasmic and membranous) was identified in 10 cases (Fig. 1f), likely reflecting the fibroblastic nature of the cells. One case showed focal EMA expression. SMA, desmin, pankeratin, MUC4 and S100 protein staining were negative in our cases.

Performing next generation sequencing 12 out of 13 specimens showed a *GNAS* mutation, these mutations were detected in in Exon 8 or 9 of the *GNAS* Gene. Four cases showed GNAS:NM_000516.5:exon8:c.C601T:p.R201C, 6 showed GNAS:NM_000516.5:exon8:c.G602A:p.R201H and 2 cases showed a mutation in GNAS:NM_000516.5:exon9:c.C679G:p.Q227E. No other non-synonymous, coding mutations were detected in the samples.

## Discussion

Precise histopathologic classification is essential for the treatment of patients with myxoid soft tumors and remains an ongoing challenge for pathologists and orthopedic tumor surgeons. Nowadays pathologists are confronted with very limited tumor material, because needle biopsies are usually performed for tumor classification before planning a surgical intervention. Highly depending on the pathologist’s diagnosis, the surgeons determine their resection margins and in case of a clear diagnosis of an intramuscular / cellular myxoma, a marginal resection would be the treatment of choice [8]. However, in case of a low-grade myxofibrosarcoma, a wide resection would be performed - since low-grade myxofibrosarcomas have the tendency to recur locally and showing an increase in tumor grade - Mentzel et al. clearly demonstrated that high-grade lesions gain the metastatic potential, they tend to metastasize in the lung, in lymph nodes, in the skin, soft tissue, or in the bone [12].

**Fig. 2:**
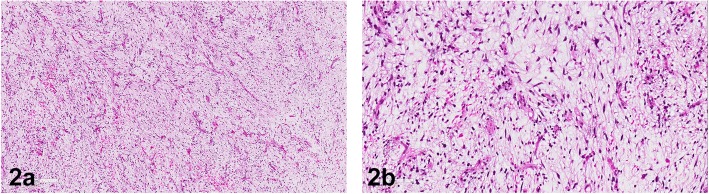
**a** Low-grade myxofibrosarcoma shows an abundant myxoid matrix with prominent curvilinear vessels and a few spindled or stellate cells with eosinophilic cytoplasm. **b** Low-grade myxofibrosarcoma shows spindled cells with hyperchromatic nuclei and eosinophilic cytoplasm mitotic figures are hardly to find

As already mentioned above, these tumors show a significant morphologic overlap and the differential diagnosis of these two lesions could be quite challenging. Both tumors show an abundant myxoid matrix and there is no helpful immunohistochemical marker to distinguish these tumors - since CD34 might be as well positive in myxofibrosarcomas in particular in superficial locations [15]. Especially in cellular myxomas curvilinear vessels can be detected, and interobserver variability occurs in the interpretation of hyperchromatic nuclei. Additionally, if muscle fibers are split up by myxoid matrix this could mimic infiltrative growth pattern. Accentuated atypical hyperchromatic spindle cells around curvilinear blood vessels are a feature of myxofibrosarcoma (Fig. 2a and b), whereas the spindle cells in cellular myxomas are more diffusely arranged in the myxoid matrix without prominent accentuation around vascular structures. Although this feature can be seen clearly in most of the resection specimens, in small biopsies these features are sometimes difficult to appreciate [4–7].

Therefore, there is a need for novel diagnostic tools that would enable precise differential diagnosis of intramuscular / cellular myxoma and low-grade myxofibrosarcoma even on biopsy material.

Other differential diagnoses of myxoid lesions, which must be considered, are low-grade fibromyxoid sarcomas, and myxoid nerve sheath tumors like neurofibromas or perineuriomas. In these cases, immunohistochemistry, fluorescence in situ hybridization (FISH) or next generation sequencing techniques facilitate in the differential diagnosis of these lesions. Low-grade fibromyxoid sarcomas show positivity for MUC4 and show a *FUS* and *CREB3L2* gene rearrangement, which can be easily demonstrated by FISH techniques. Neurofibromas show S100 positivity by immunohistochemistry, while perineuriomas express EMA at least focally [2, 3].

Okamoto et al. first described *GNAS* mutations in intramuscular / cellular myxomas in 2000 [16]. This gene is located on chromosome 20q13.2-q13.3, harbors 13 exons, shows alternative splicing and is involved in the genomic imprinting [6]. This gene encodes for the alpha-sub-unit of the heterotrimeric G-protein and it is involved in cell signaling and leads to transcription of the protein c-Fos and in the activation of the cell cycle. Additionally, *GNAS* mutations and c-Fos are also involved in the pathogenesis of fibrous dysplasia. This is a benign bone tumor associated with intramuscular myxoma in Mazabraud syndrome [7, 17].

By reviewing the literature Willems et al. found *GNAS* mutations in 50% intramuscular / cellular myxomas [7]. Delaney et al. found in 29–61% cases of intramuscular myxomas a *GNAS* mutation depending on the technique (29% using conventional PCR followed by mutation-specific restriction enzyme digestion and 61% mutations were detected by using COLD-PCR/MSRED [5]. Walther et al. described *GNAS* mutations in 36.5% of intramuscular myxomas [6]. In all these 3 studies low-grade myxofibrosarcomas did not show a *GNAS* activating mutation [5–7]. Additionally, Heitzer et al. showed in a recent publication that myxofibrosarcomas did not demonstrate *GNAS* mutations. However, their study showed somatic copy number alterations (SCNA) irrespectively of the tumor grade [18].

## Conclusion

In summary we performed mutational analysis by next generation sequencing in 13 specimens. Twelve cases out of 13 (92.3%) showed a *GNAS* mutation. Therefore, our data highly suggest that more intramuscular / cellular myxomas show a *GNAS* mutation than initially reported in the literature. As nearly all mutations of *GNAS* are located at R201 or Q227 it would also be feasible to use pyrosequencing or mutation specific qPCR to detect *GNAS* mutations in a diagnostic lab not equipped with next generation sequencing methods.

In conclusion, we strongly suggest that next generation sequencing techniques facilitate in the diagnosis of myxoid soft tissue tumors, especially on small biopsy specimens. We were able to demonstrate that the detection of *GNAS* mutations is more common in intramuscular / cellular myxoma than reported in the past. *GNAS* mutations can be used as good diagnostic tool to distinguish intramuscular / cellular myxoma from low-grade myxofibrosarcoma, especially on biopsy material.

Exact classification of myxoid soft tissue tumors on small biopsies by using next generation sequencing techniques in combination with HE morphology and immunohistochemistry enables adequate treatment.

## Abbreviations

FISH: Fluorescence in situ hybridization
HE: Hematoxylin and eosin
PCR: Polymerase chain reaction
PCR-MSRED: polymerase chain reaction mutation-specific restriction enzyme digestion
qPCR: Real time polymerase chain reaction
SCNA: Somatic copy number alterations
